# Mesenchymal cells from limbal stroma of human eye

**Published:** 2008-03-04

**Authors:** Naresh Polisetty, Anees Fatima, Soundarya Lakshmi Madhira, Virender Singh Sangwan, Geeta K Vemuganti

**Affiliations:** 1 Sudhakar Sreekanth Ravi Stem Cell Biology Laboratory, LV Prasad Eye Institute, Kallam Anji Reddy Campus, Hyderabad, India; 2 Cornea and Anterior Segment Service, LV Prasad Eye Institute, Kallam Anji Reddy Campus, Hyderabad, India

## Abstract

**Purpose:**

Mesenchymal stem cells (MSC) are self-renewing, multipotent cells that are present in many adult tissues, including bone marrow, trabecular bone, adipose, and muscle. The presence of such cells of mesenchymal origin and their role during the wound healing of ocular injuries are currently being explored by many studies worldwide. In this study, we aimed to report the presence of mesenchymal cells resembling bone marrow-derived cells (MSC-BM) in the limbus of the human eye.

**Methods:**

Fresh limbal tissues obtained from human subjects undergoing limbal biopsy for ocular surface reconstruction were used to establish limbal mesenchymal cell (MC-L) cultures. The spindle cell outgrowths observed in extended limbal epithelial cultures (LECs) and from deepithelialized limbal tissues were serially passaged using a human corneal epithelial (HCE) medium, which contained epidermal growth factor (EGF) and insulin, and supplemented with fetal bovine serum (FBS). MSC cultures were established from human bone marrow samples using Dulbecco’s Modified Eagles Medium (DMEM) supplemented with FBS. The mesenchymal cells from both extended limbal cultures (MC-L) and bone marrow (MSC-BM) were characterized by morphology and immunophenotyping using epithelial, mesenchymal, hematopoietic, and endothelial markers using fluorescent-activated cell sorting (FACS). Selective markers were further confirmed by immunostaining and reverse transcription polymerase chain reaction (RT–PCR). Stromal cells of both origins (limbal and bone marrow-derived) were also evaluated for colony forming ability and population doubling. Attempts were made to differentiate these into adipocytes and osteocytes using conditioned medium.

**Results:**

Spindle cells from extended limbal epithelial cultures as well as de-epithelialized human limbal tissues appeared elongated and fibroblast-like with oval vesicular nuclei. Both MC-L and MSC-BM showed colony forming ability in 14 days of plating. MC-L showed a population doubling of 22.95 while in MSC-BM, it was 30.98. Immunophenotyping of these cells by FACS and immunocytochemistry showed that the MC-L were positive for mesenchymal markers and negative for epithelial and hematopoietic markers similar to MSC-BM. The MC-L phenotype has thus been defined as MC-L^CD105, CD106, CD54, CD166, CD90, CD29, CD71, pax −6 +/ p75, SSEA1, Tra-1–61, Tra-1–81, CD31, CD34, CD45, CD11a, CD11c, CD14, CD138, Flk1, Flt1, VE-Cadherin -^. The profile was further confirmed by RT–PCR. These cells also showed differentiation into adipocytes and osteocytes.

**Conclusions:**

We demonstrated the presence of mesenchymal cells in the human limbus, similar to the bone marrow-derived MSC-BM. This presence suggests that these cells are unique to the adult stem cell niche.

## Introduction

The limbus of the eye, located at the junction of the cornea and conjunctiva of the ocular surface, is now extensively used for ocular surface resurfacing in patients with limbal stem cell deficiency (LSCD) [1-3]. It is now established that the progenitor cells that regenerate corneal epithelium reside in the limbus [4,5]. In addition to this regenerative capacity, limbal epithelial cells have also been reported to have features of “plasticity,” evident from the neuronal-like differentiation of these cells [6,7]. A recent report by Dravida et al. [8] points to the presence of fibroblast-like cells in the limbal stroma, possessing a stem cell-like self-renewal property with plasticity. However, such cells have not been reported from human limbal tissues. We had observed the presence of spindle cell outgrowths in late limbal epithelial cultures, which were non-epithelial in nature. These cultures were serially passaged and characterized for surface markers.

Mesenchymal stem cells or bone marrow stromal cells (MSC-BM) are multipotent stem cells with high self-renewing capacity and the ability to differentiate into more than two lineages in vitro or in vivo into osteoblasts, chondrocytes, myocytes, adipocytes, beta-pancreatic islets cells, or neuronal cells. MSC-BM were earlier believed to nurture the hematopoietic stem cells by releasing Granulocyte colony-stimulating factor (GCSF), cytokines, etc. These differentiated cells do not express hematopoietic and endothelial markers (such as CD45, CD11c, and CD31) but express mesenchymal markers, CD90, SH2 (endoglin or CD105), SH3, or SH4 (CD73 and STRO-1) [9]. MSC-BM have been isolated by means of rapid expansion in serum-containing media and adherence, from several tissues including bone marrow, amniotic fluid, peripheral blood, adipose tissue, dermis, articular synovium, compact bone, muscle, and brain [10,11]. In response to specific culture conditions, these cells can give rise to multiple mesenchymal-derived cell types such as osteoblasts [12], chondrocytes [13], adipocytes [14], myloblasts [15], and neural cells [16]. In this paper, we reproduced the properties of mesenchymal stem cells as rapidly adhering marrow stromal cells with the ability to form colonies and differentiate into different cell types such as osteocytes and adipocytes. We aimed to investigate if the limbal spindle cells were of mesenchymal origin (MC-L) by comparing them with human MSC-BM both in terms of immunophenotype and plasticity.

## Methods

The protocol was approved by our Institutional Review Board and the research followed the tenets of the Declaration of Helsinki.

### Establishment of cell cultures

Limbal tissues were obtained with informed consent from patients undergoing limbal biopsy for cultivated limbal epithelial transplantation. The surgical procedure included careful dissection of a 1 × 2 mm^2^ piece of limbal epithelium with 0.5 mm depth, originating 3 mm behind the limbus and extending into clear corneal stromal tissue at the limbus under strict aseptic conditions. The tissue was transported in a human corneal epithelium (HCE) medium to the tissue culture laboratory where limbal epithelial cultures were established on de-epithelialized human amniotic membrane (HAM) with the basement side up using our previously reported protocol [2]. While cultures with a monolayer of epithelial cells growing from the explants in 10–14 days were terminated for transplantation, parallel plates were cultured further for two to three weeks when spindle cell-like outgrowths were seen under a phase contrast microscope (Olympus, Tokyo, Japan). These were then trypsinized and plated on a T25 flask. After two days of plating, we observed adherent spindle cells called limbal mesenchymal cells. Residual epithelial cells were removed by changing the medium. Adherent MC-L were cultured in HCE medium supplemented with 10% fetal bovine serum (FBS; Sigma-Aldrich Chemie, Steinheim, Germany). The cultures were maintained in 5% CO_2_ in a humidified incubator at 37 °C. When the cells reached 80%–90% confluency, cultures were harvested with 0.25% trypsin (Sigma Chemical Co., St.Louis, MO) and 1 mM EDTA solution (Sigma) from passages P0 through P6.

**Table 1:** Primers used in reverse transcription polymerase chain reaction.

Primer	Primer sequence	Product size	Annealing temperatures
ΔNp63α	F: GGAAAACAATGCCCAGACTC	1387 bp	60 °C
	R: ATGATGAACAGCCCAACCTC		
Cytokeratin K3	F: GGCAGAGATCGAGGGTGTC	150 bp	60 °C
	R: GTCATCCTTCGCCTGCTGTAG		
Cytokeratin K12	F: ACATGAAGAAGAACCACGAGGATG	150 bp	60 °C
	R: TCTGCTCAGCGATGGTTTCA		
α-Enolase	F: GTTAGCAAGAAACTGAACGTCACA	619 bp	60 °C
	R: TGAAGGACTTGTACAGGTCAG		
Pax−6	F: ATAACCTGCCTATGCAACCC	208 bp	55 °C
	R: GGAACTTGAACTGGAACTGAC		
Connexin 43	F: CCTTCTTGCTGATCCAGTGGTAC	145 bp	60 °C
	R: ACCAAGGACACCACCAGCAT		
EGFR	F: TCTCAGCAACATGTGGATGG	474 bp	60 °C
	R: TCGCACTTCTTACACTTGCG		
p75	F: TGAGTGCTGCAAAGCCTGCAA	230 bp	55 °C
	R: TCTCATCCTGGTAGTAGCCGTAG		
Integrin a9	F: TGGATCATCGCCATCAGTTTG	123 bp	55 °C
	R: CCGGTTCTTCTCAGCTTCGAT		
GAPDH	F: GCCAAGGTCATCCATGACAAC	498 bp	54.2 °C
	R: GTCCACCACCCTGTTGCTGTA		

The table details the primers and sequences, their expected product size and the annealing temperatures used in polymerase chain reaction

To confirm the origin of these spindle cells from the limbus, we also grew spindle cells from de-epithelialized limbal tissues. Limbal tissues were de-epithelialized using dispase (BD Biosciences, Mississauga, Canada) at a concentration of 1.2 U/ml, digested with trypsin-EDTA to make a single cell suspension, and then plated on the T25 flasks. At confluence, cells were trypsinized and passaged as above.

For comparative analysis, MSC-BM were isolated by the Ficoll-Hypaque (Sigma Chemical Co., St.Louis, MO) density gradient method and cultured on the basis of adherent properties. Briefly, human MSC cultures were established from five bone marrow aspirates of healthy donors after obtaining informed consent. The bone marrow mononuclear cells (BMMNCs) were separated using Ficoll-Hypaque gradient at 400x g for 30 min. The mononuclear cells were then plated at a density of 1x10^7^ cells in Dulbecco's Modified Eagle's Medium (DMEM; Sigma-Aldrich Chemie, Steinheim, Germany) supplemented with 10% FBS. When cultures reached confluence, cells were passaged using trypsin-EDTA.

### Colony-forming unit assays

For these assays, cells of both origins (MC-L and MSC-BM) were plated at two cells per cm^2^ and cultured for 14 days in 75 cm^2^ tissue culture flasks. After 14 days, the cultures were stained with 0.5% crystal violet in methanol for 5 min. The colony count was performed and colonies that were less than 2 mm in diameter or faintly stained were excluded.

**Figure 1:**
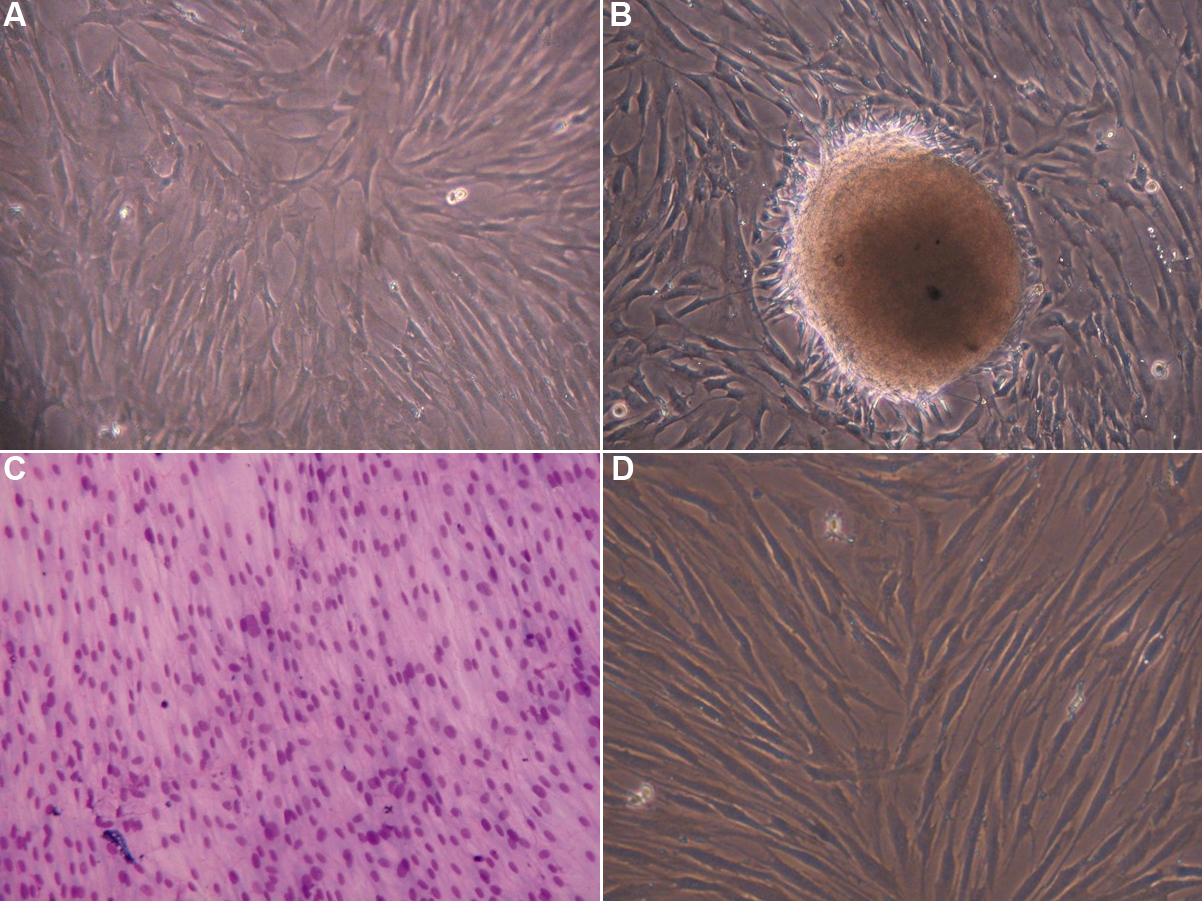
Morphology of cultured mesenchymal cells from limbus (MC-L) and mesenchymal stem or stromal cells from bone marrow (MSC-BM). The phase contrast microscopic picture of MC-L shows the spindle morphology (magnification: 200X) (**A**); Cell sphere formation in the MC-L cultures gives the impression of embryoid body formation (magnification: 200X) (**B**); spindle shaped morphology of MC-L as confirmed by Giemsa stain (Light microscope, magnification: 200X) (**C**); Culture of MSC-BM (magnification: 200X) (**D**) showing spindle cell morphology similar to that of MC-L.

### Population doublings

Population-doubling assay was performed on MSC-BM from passage 1–5 and on MC-L from passages 2–6. Passages 1 and 2 were not included for MC-L as mesenchymal cells derived from cultured limbal epithelial cells had epithelial cell contamination. Cells from MC-L and MSC-BM were seeded (1 × 10^4^ cells) at each passage and trypsinized after 10 days and 12 days, respectively. The population doubling of cells was calculated as:

Number of Cell Doublings (NCD)=log _10_(y/x)/log _10_2,

Where “y” is the final density of the cells and ‘x’ is the initial seeding density of the cells.

**Figure 2:**
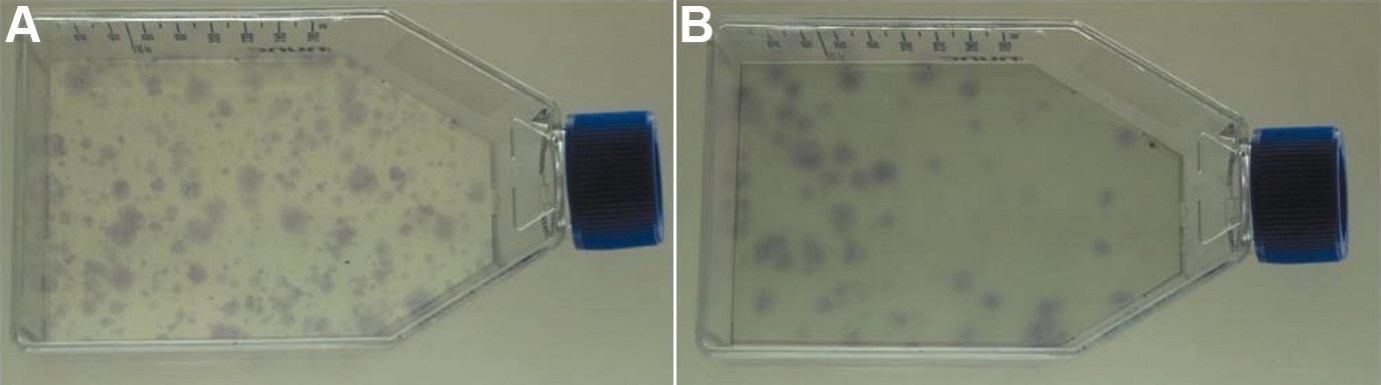
Colony Formation Unit (CFU) assay of MC-L and MSC-BM. The figure shows the crystal violet stained colonies of stromal cells – MC-L (**A**) and MSC-BM (**B**) in T75 flasks.

### Characterization of limbal mesenchymal cells and MSC-BM

#### Flow cytometry

MC-L and MSC-BM were characterized for a battery of mesenchymal (CD90, CD105, CD29, CD71, CD166), epithelial (K3, K14), hematopoietic (CD34, CD45, CD11a, CD11c, CD138, CD68, CD25, CD4, CD8, CD14), embryonic (SSEA1, TRA-1–60, TRA-1–81), and endothelial makers (VCAM, ICAM, VEGF receptor [Flk1], Flt-1, PECAM [CD31], VE-cadherin) by fluorescent-activated cell sorting (FACS). Briefly, a single cell suspension (0.5 to 1x 10^6^ cells each) of MC-L and MSC-BM at passage 2, in 100 µl of buffer (phosphate buffered saline, [PBS]; 0.1% sodium azide; 2% FBS), was incubated with saturating concentrations of respective primary antibodies for 40 min. After three washes, the cells were centrifuged at 200x g for 5 min, resuspended in ice-cold PBS, and then incubated at 4 °C with the FITC-labeled secondary antibody for 30 min in the dark. Cell fluorescence was evaluated by flow cytometry in FACS caliber instrument (Becton Dickinson; BD, Heidelberg, Germany), and FACS aria (BD, San Jose, CA) and data were analyzed by using Cell Quest software (BD, San Jose, CA). An isotype control was included in each experiment and specific staining was measured from the cross point of the isotype with a specific antibody graph. A total of 10,000 events were acquired to determine the positivity of different cell surface markers used.

#### Immunocytochemistry

The expression of selected markers was further confirmed by immunocytochemistry using vimentin, CD90, CD34, CD45, CD11a, CD11c, CD25, CD29, K3, and K14. MC-L and MSC-BM at passage 2 were seeded into 24 well plates and cultured to confluency. Cells were then fixed with 70% alcohol or 4% paraformaldehyde in 0.1 M phosphate buffer (pH 7.2) for 20 min and processed for immunocytochemistry. Non-specific reactions were blocked with 5% FCS for 30 min at room temperature. Fixed cells were then incubated for 1 h with primary antibodies, detected using FITC-conjugated secondary antibody and counterstained with propidium iodide (PI). The stained preparations were screened with a laser scanning confocal microscope (LSM510; Carl Zeiss Inc., Thornwood NY) using a fluorescent light source (excitation wavelength 480 and 540 nm).

**Table 2:** Population doublings of cultured MC-L from P0 through P6.

Passage number	Initial cell density	Incubation time (Days)	Mean final cell number (x millions)	Number of cell doublings (NCD)	Accumulative NCD	Population doubling time (h)
P0		23				
P1		3–4	1.8			
P2	1×10^4^	10	3.0	8.2295	8.2295	29.1633
P3	1×10^4^	10	1.3	7.0230	15.2525	34.1734
P4	1×10^4^	10	0.13	3.7008	18.9533	64.8508
P5	1×10^4^	10	0.08	3.0002	21.9535	79.9946
P6	1×10^4^	10	0.02	1.000	22.9535	240

The table summarizes the results of population doubling study of cultured MC-L from passages 0 (P0) to 6 (P6), indicating the initial cell density, mean final cell number and the number of cell doublings calculated using the formula. Passages P0 and P1 have not been included for calculations due to epithelial cell contamination.

**Table 3:** Population doublings of cultured MSC-BM from P0 through P5.

Passage number	Initial cell number	Incubation time (Days)	Mean final cell number (X millions)	Number of Cell Doublings (NCD)	Accumulative NCD	Population doubling time (h)
P0	1×10^7^	13	1.2			
P1	1×10^4^	13	1.5	7.2295	7.2295	33.1973
P2	1×10^4^	13	1.4	7.1299	14.3594	33.6610
P3	1×10^4^	13	1.4	7.1299	21.4893	33.6610
P4	1×10^4^	13	0.72	6.1705	27.6598	38.8947
P5	1×10^4^	13	0.1	3.322	30.9820	72.2412

The table summarizes the results of population doubling study of cultured MC-L from passages 0 (P0) to 6 (P6), indicating the initial cell density, mean final cell number and the number of cell doublings calculated using the formula.

### Reverse transcription polymerase chain reaction analysis

Selective epithelial and corneal cell type-related marker expression in MC-L and MSC-BM was studied by reverse transcription polymerase chain reaction (RT–PCR). Both the stromal cell types were evaluated for epithelial stem-cell related markers - p63α and integrin α9; PAX-6 which is selectively expressed by cells of neuroectodermal origin and during ocular development; corneal epithelium-related cytokeratin pair K3/K12; growth factor receptors - epidermal growth factor receptor (EGFR) and low affinity nerve growth factor (NGF) receptor p75; gap junction protein connexin 43 and glycolytic enzyme α-enolase. Limbal epithelial cells (LECs) were also tested for these markers simultaneously. Expression of PAX−6 was studied to confirm the origin/resemblance of MC-L to mesenchymal or epithelial cells.

Total RNA was extracted from two- to three-week-old limbal epithelial cultures and MC-L and MSC-BM at passage 2 using Trizol^TM^ (Invitrogen, Carlsbad, CA) according to the manufacturer’s protocol. The extracted RNA was quantified by reading the absorbance at 260 nm, and its purity evaluated from the 260/280 ratio of absorbance in spectrophotometer (Model UV-1601; Shimadzu Corporation, Koyto, Japan). This RNA (4 μg) was used for cDNA preparation using murine leukemia virus reverse transcriptase (#EP0451; MBI Fermentas, Vilnius, Lithuania) and was subjected to a semi-quantitative PCR with primers (Table 1) at initial denaturation of 94 °C for 3 min, denaturation at 94 °C for 1 min, extension of 72 °C for 1 min, and a final extension of 10 min at 72 °C for a total of 40 cycles. The PCR products were analyzed on a 1.2% agarose gel and scanned using an ultraviolet (UV) gel doc (UVtec Ltd, Cambridge, UK). The expression of various markers was normalized using GAPDH as an internal control.

### Differentiation

#### Adipogenic differentiation

Passage 2 cells (both MSC-BM and MC-L) were seeded on coverslips in six-well plates and cultured in a complete medium to confluency. At confluency, the cells were switched to an adipogenic medium (DMEM/10% FBS supplemented with 0.5 µM dexamethasone, 0.5 mM isobutylmethylxanthine, and 10 µg/ml insulin, Sigma Chemical Co., St.Louis, MO) and further cultured for 21 days with the medium being changed on every alternate day. After 21 days, the adipogenic cultures were fixed in 4% paraformaldehyde for at least 1 h and stained with fresh 0.3% oil Red-O solution for 2 h. After staining, the cultures were washed three times and counterstained with hematoxylin.

#### Calcification

Passage 2 cells (both MSC-BM and MC-L) were seeded on coverslips in six-well plates and cultured in a complete medium to confluency. The medium in the culture was then replaced with a calcification medium containing DMEM/10% FBS, 100 nM dexamethasone, 10 mM β-glycerophosphate, and 50 µM ascorbic acid (Sigma Chemical Co., St.Louis, MO) and incubated for 21 days. These coverslips were stained with fresh 0.5% alizarin red solution.

## Results

Spindle cell cultures were established from both extended limbal epithelial cultures and de-epithelialized limbal tissues. Under a phase contrast microscope the cells appeared fibroblastic, elongated, and spindle shaped with a single nucleus (Figure 1A). These cells showed the ability to form colonies with the occasional cell sphere formation giving an impression of embryoid bodies (Figure 1B). The fibroblastic morphology was confirmed by Giemsa staining (Figure 1C).

Spindle shaped MSC-BM were established from patients’ unstimulated bone marrow specimens. The cells appeared spindle shaped with a single nucleus under phase contrast microscope (Figure 1D).

### Colony forming unit assay

When plated at 2 cells/cm^2^, MC-L in culture showed a colony forming efficiency between 30%–40% at passage 2 (Figure 2A), 10%–15% at passage 3 (P3), and 8% at P4 while MSC-BM showed a colony forming unit (CFU) of 20% at passage 2 (Figure 2B), 8%–12% at passage 3, and 2%–4% at passage 4. At P5, the cells showed no colony forming ability illustrating that the colony forming ability decreases with increasing passages.

### Population doubling assay

While MC-L showed 22.95 population doublings, MSC-BM showed 30.98 cell doublings. The results of this assay are summarized in Table 2 and Table 3, which show the population doublings from passages 0–6.

**Table 4:** Surface antigen profile of MC-L and MSC-BM.

Serial number	Marker	MC-L	MSC-BM
1	CD 34	- (1.03±0.4)	- (0.19%±0.02)
2	CD45	- (0.95%±0.43)	- (0.89%±0.2)
3	CD11a	- (0.28%±0.1)	- (0.87%±0.2)
4	CD11c	- (0.0%)	- (0.0%)
5	CD138	- (0.98%±0.2)	- (1.35%±0.5)
6	CD106/VCAM	+ (50.0% ±5.57)	+ (54.67% ± 5.86)
7	CD105	+ (21.42% ± 4.133)	+ (71.33% ± 6.66)
8	CD90	+ (95.63% ± 2.11)	+ (94.57% ± 2.00)
9	CD29	+ (86.33% ± 3.06)	+ (84.0% ± 2.65)
10	CD71	+ (66.07% ± 2.57)	+ (45.27% ± 4.15)
11	HLA-ABC	+ (93.44% ± 4.32)	+ (91.33% ± 2.75)
12	HLA-DR	- (0.67%±0.1)	- (0.87%±0.14)
13	CD4	- (0.99%±0.13)	- (0.15%±0.09)
14	CD8	- (0.78%±0.15)	- (0.66%±0.12)
15	K3	- (0.67%±0.27)	
16	K14	- (0.95%±0.2)	
17	CD 68	- (0.84%±0.43)	- (0.76%±0.025)
18	ICAM/CD54	+ (28.13% ± 4.01)	+ (24.0% ± 4.58)
19	CD166	+ (81.67% ± 3.51)	+ (83.67% ± 2.08)
20	CD31	- (0.45± 0.23)	- (0.65% ±0.37)
21	CD14	+ (1.2%±0.43)	+ (1.4%±0.5)
22	SSEA1	- (0.00%)	
23	TRA-1–61	- (0.00%)	
24	TRA-1–81	- (0.00%)	
25	VE-Cadherin	- (0.00%)	
26	Flk1	- (0.00%)	
27	Flt1	- (0.00%)	
28	CD25	- (0.64±0.24)	- (0.45±0.12)

The table summarizes the results of immunophenotyping of MC-L and MSC-BM by FACS Analysis for epithelial, mesenchymal, endothelial and embryonic markers. Percentage of expression of a marker are given within brackets (average values of three such experiments ± standard deviation. The symbol (–) indicates the negative expression for a marker while the symbol (+) indicates the positive expression of a marker.

### Characterization of limbal mesenchymal cells and MCS-BM

#### Flow cytometry

**Figure 3:**
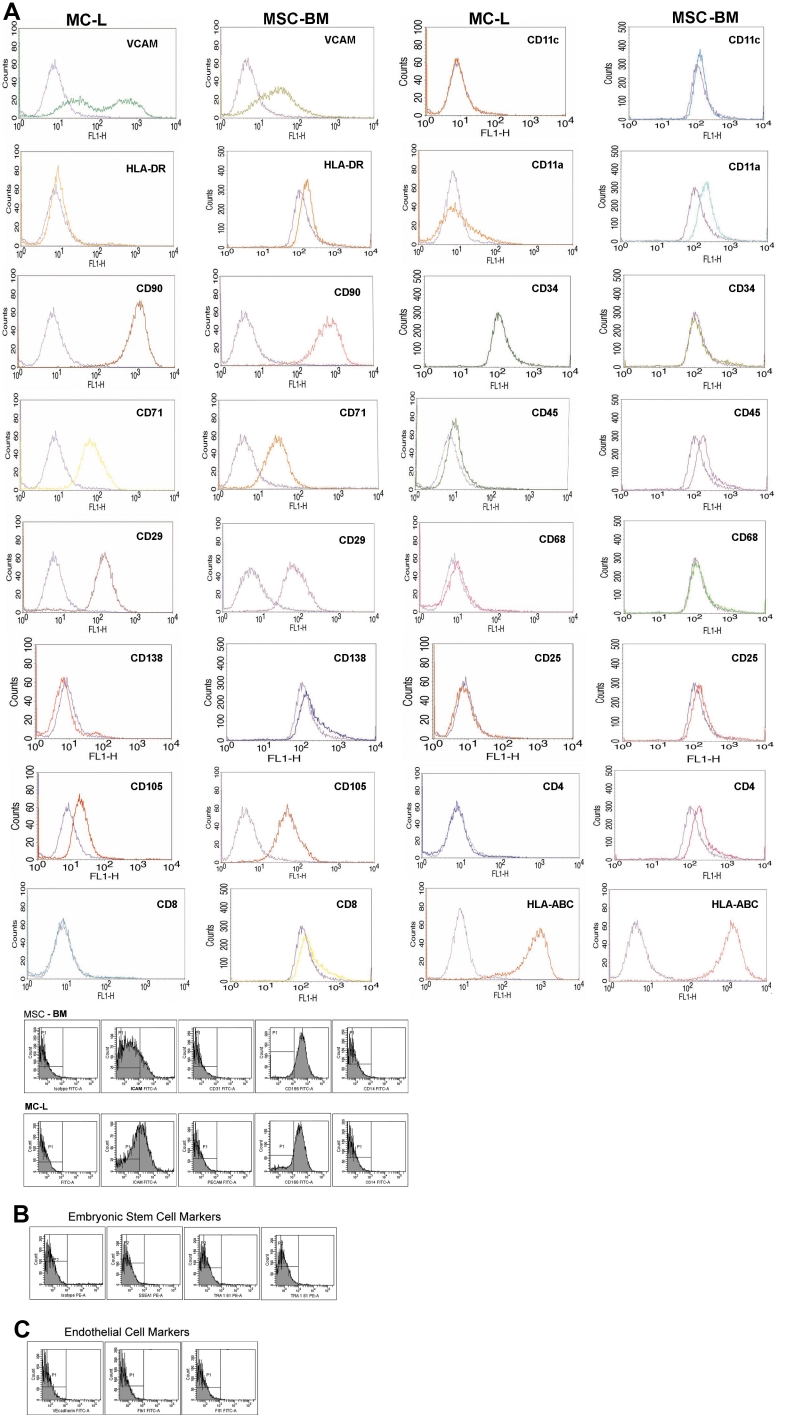
Surface marker profile of MC-L and MSC-BM by FACS analysis. FACS analysis of MC-L cells and MSC-BM for a battery of markers shows a close resemblance of MC-L toward mesenchymal phenotype as that of MSC-BM (**A**) (The purple line in the histograms represents the isotype control). FACS analysis of MC-L for expression of both embryonic stem cell markers (**B**) and endothelial markers (**C**) is also shown to be negative.

FACS analysis revealed similarities in surface marker expression of MC-L with MSC-BM (Figure 3A). Table 4 summarizes the surface marker expression profile of cultured MC-L and MSC-BM. The cells have shown no expression of embryonic markers (Figure 3B) or other endothelial markers (Figure 3C).

#### Immunocytochemistry

**Figure 4:**
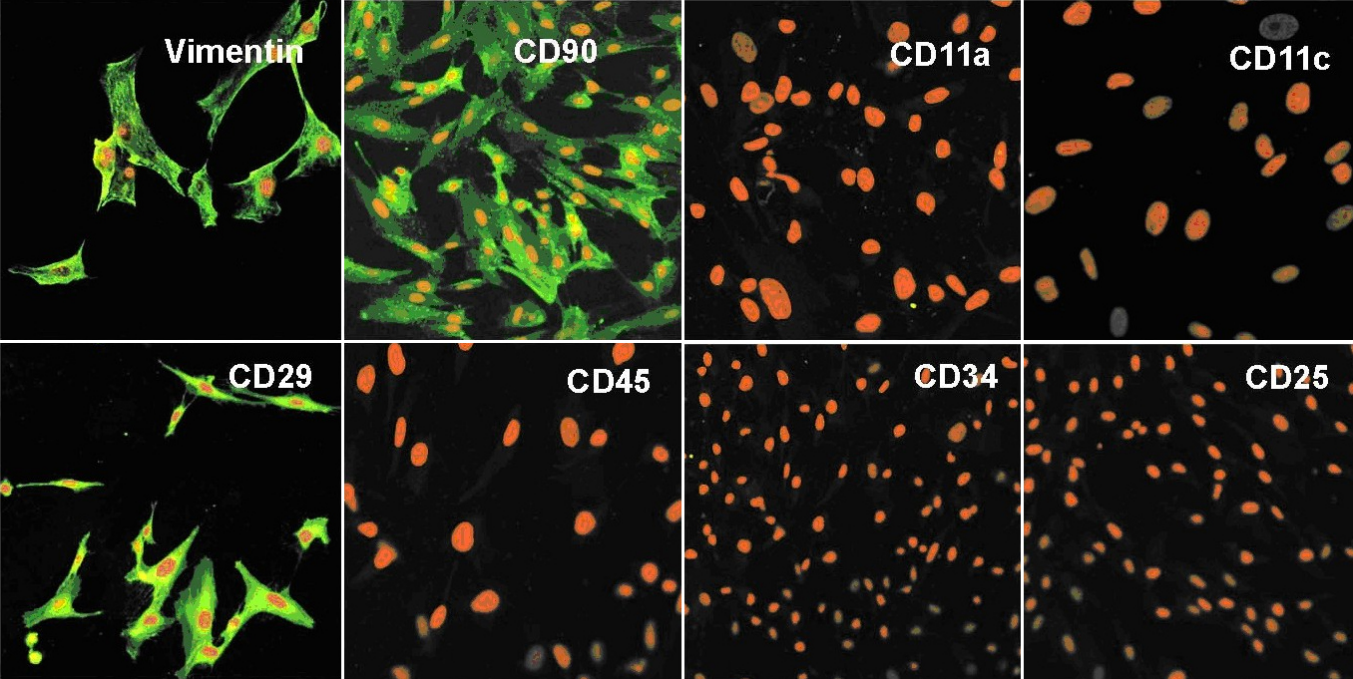
Mesenchymal phenotype of MC-L by Laser Scanning Confocal Microscopy. LSCM pictures of MC-L show positivity (green fluorescence) for vimentin (20X), CD90 (20X), and CD29 (20X) and negativity for CD11c (40X), CD11a (40X), CD45 (40X), CD34 (20X), and CD25 (20X). The nuclei are counterstained with propidium iodide (red).

**Figure 5:**
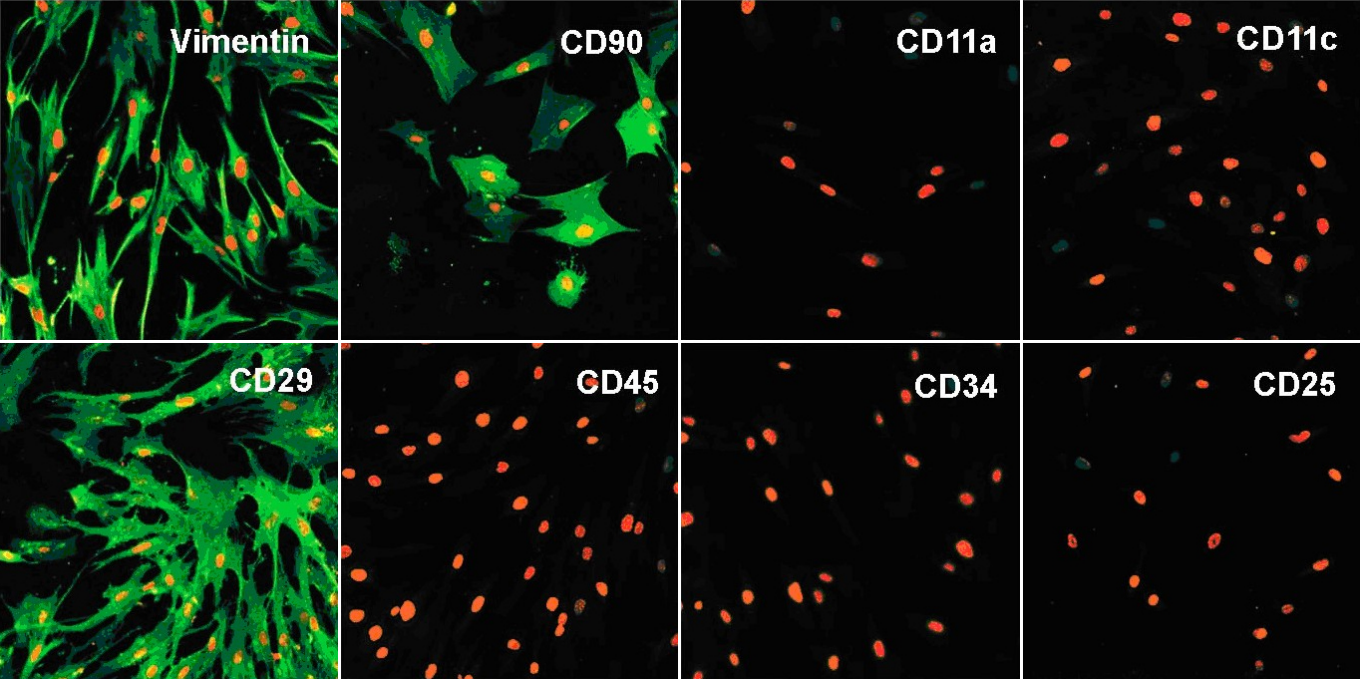
Mesenchymal phenotype of MSC-BM by Laser Scanning Confocal Microscopy. LSCM pictures of MSC-BM show positivity for vimentin (20X), CD90 (20X), CD29 (20X) and negativity for CD11c (20X), CD11a (20X), CD45 (20X), CD34 (20X), and CD25 (20X). The nuclei are counterstained with propidium iodide (red).

**Figure 6:**
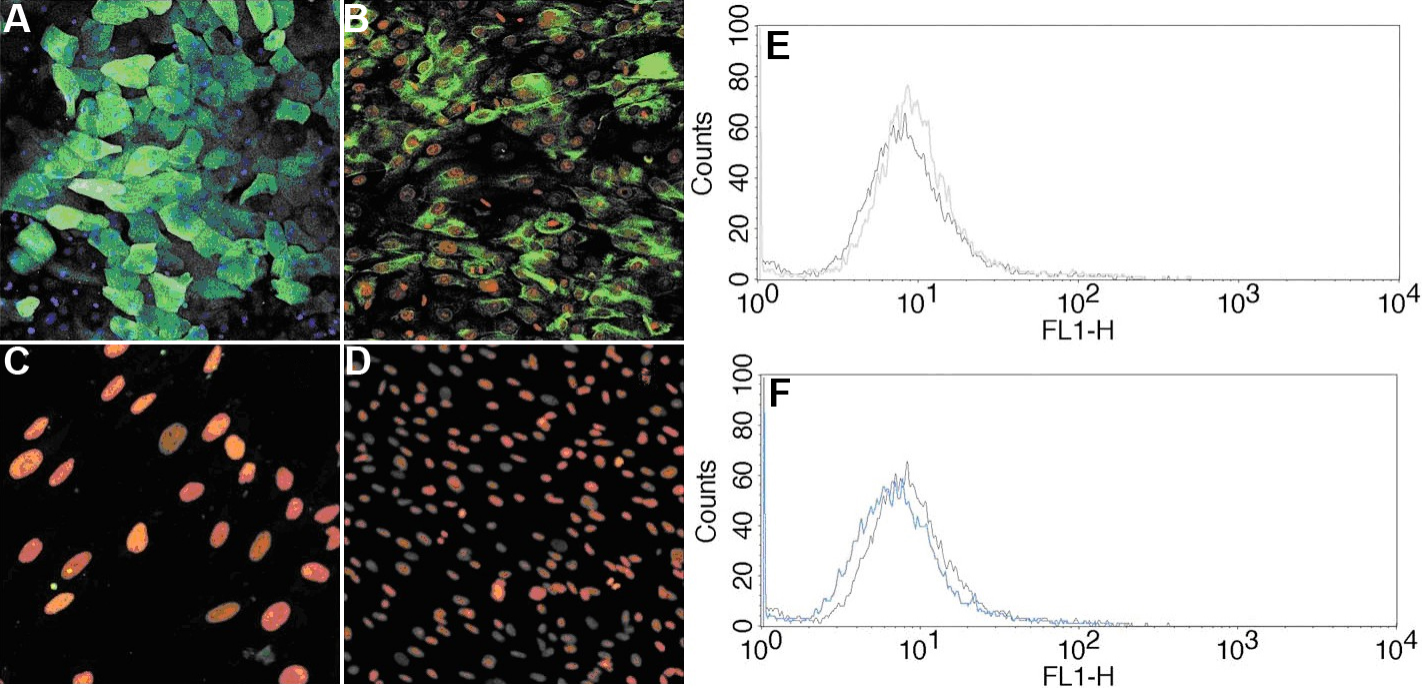
Epithelial phenotype of LEC and MC-L by Laser Scanning Confocal Microscopy and FACS analysis. LSCM pictures of limbal epithelial cells (LECs) show positivity for K3 (20X) and K14 (20X; **A,B**), and LSCM pictures of MC-L show negativity for K3 (40X) and K14 (20X; **C,D**). The nuclei are counterstained with propidium iodide (blue, red). FACS histograms of MC-L confirm the absence of K3 (**E**) and K14 (**F**) expression. The blue line represents the test sample and the purple line represents the isotype control.

Upon immunostaining, MC-L were positive for mesenchymal markers - CD90, CD29, and vimentin, and negative for hematopoietic markers (Figure 4). MSC-BM showed a similar expression profile (Figure 5). The MC-L also showed negative staining for epithelial markers, K3 and K14 (Figure 6).

### Reverse transcription polymerase chain reaction analysis

**Figure 7:**
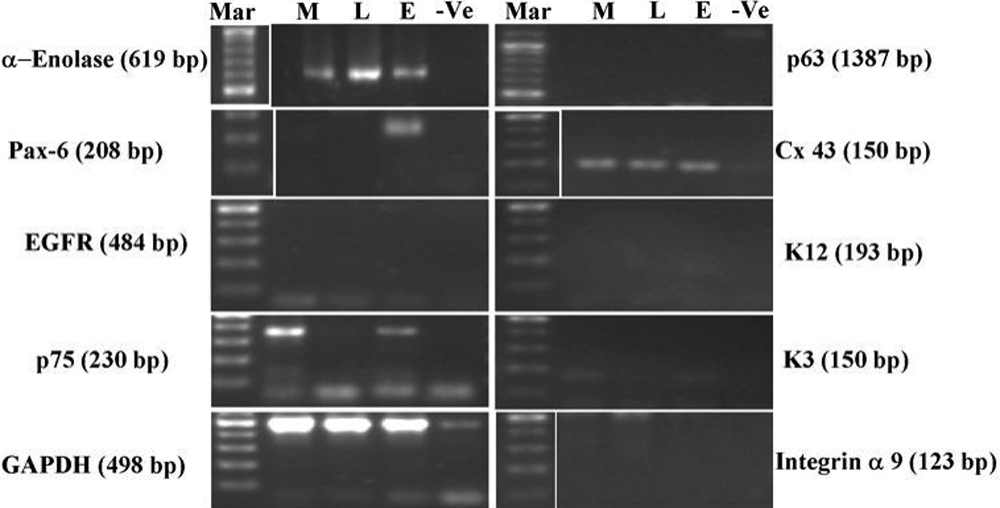
Reverse transcription polymerase chain reaction analysis of limbal mesenchymal cells and MSC-BM. The figure shows the expression profiles of selected markers in MSC-BM (M), MC-L (L), and LECs (E) as well as the negative control (-ve). MSC-BM and MC-L show negative expression profiles of p63 (1387 bp), corneal cytokeratins, K3 (150 bp) and K12 (193 bp), integrin α9 (123 bp), EGFR (484 bp), and PAX−6 (208 bp) in comparison to LECs. The figure also shows a positive expression of connexin 43 (150 bp) and α-enolase (619 bp) by stromal cells of both origin similar to LECs and an expression of p75 by MSC-BM and LECs (230 bp). The above expression studies have been normalized using GAPDH (498 bp) as the internal control.

RT–PCR analysis of MC-L and MSC-BM showed similarities in expression profiles of various epithelial markers (Figure 7). Stromal cells of both origins showed no expression of epithelial markers such as p63, EGFR, PAX−6, integrin α9, and corneal cytokeratins K3/K12 while there was an expression of common cellular markers such as α-enolase and connexin 43 as compared to limbal epithelial cells. Also, the MC-L showed a negative expression of NGF receptor p75, while the MSC-BM showed a positive expression.

### Differentiation

**Figure 8:**
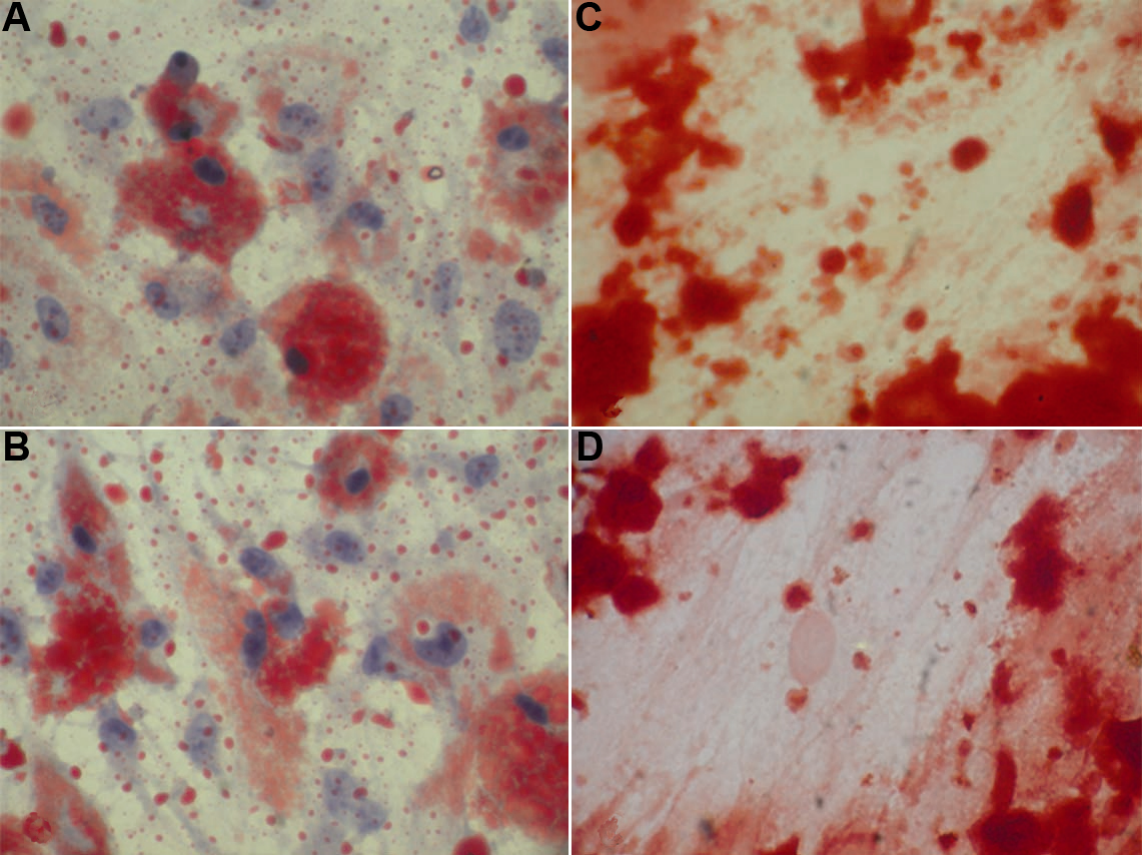
Differentiation potential of MC-L and MSC-BM into adipocytes and osteocytes. Adipocyte differentiation of stromal cells shows the presence of oil-red positive lipid laden cells in MSC-BM (**A**) and MC-L (**B**). Osteogenic differentiation of these cells shows the presence of alizarin stained calcium deposits in MSC-BM (**C**) and MC-L (**D**) indicating calcification of cells. All these images are at a magnification of 20X.

MSC-BM and MC-L were differentiated in vitro using adipogenic and osteogenic induction media. Three weeks after the adipogenic induction, the cells stained oil red O positive, which meant the cells were showing a lipid laden adipocyte phenotype. (Figure 8A,B). Similarly, these cells induced with osteogenic induction for two to three weeks showed calcification when stained with alizarin red for calcium deposits (Figure 8C,D).

## Discussion

Limbal epithelial cells are cultivated in vitro and used for clinical applications in patients with LSCD in many centers worldwide, including ours [1-3]. We made an interesting observation that when in culture for a longer time, these epithelial cells give rise to fibroblast-like cells. We speculated that these cells were of stromal or mesenchymal origin as they had a longer initial lag phase in comparison to limbal epithelial cells in culture. The presence of similar cells in de-epithelialized tissue further pointed toward their stromal or mesenchymal origin. This is similar to the observations made by Dravida et al. [8] who showed the existence of fibroblast-like cells in the limbal stroma with embryonic stem (ES) cell-like features. Another study by Funderburgh et al. [17] evaluated the stromal cells isolated from the corneal stroma with features of stem cells as proved by ABCG2 and PAX−6 expression and side population studies. They also demonstrated that the location of such cells was more toward the limbus than the central cornea.

Cultured human corneal epithelial stem cells from the limbus have been successfully used for corneal reconstruction. The phenotypic characterization of these stem cells has been well documented. This population of MC-L is different from the above-mentioned limbal epithelial stem cells (LESCs) in their origin. Limbal epithelial stem cells are cultured from the corneo-limbal epithelium [18] while isolated and cultured limbal stromal cells (LSC; from extended limbal epithelial cultures) were adherent to the plastic surface. Morphologically, LESCs were relatively small and cuboidal with 10.1 μm in diameter [19], structurally and biochemically primitive with low cellular granularity, and pigmented. On the other hand, MC-L were characteristically elongated and spindle shaped. LESCs are shown to possess label retaining property and to express α-enolase, vimentin, cytokeratin 3, cytokeratin 14, and p63α, a transcription factor, whereas MC-L do not express epithelial markers such as p63α and cytokeratins 3 and 14.

There is sufficient work done on the human MSC-BM from their characterization and differentiation to clinical application. Human MSC-BM are currently being tested in several animal models for human diseases [20,21], and several clinical trials making use of these cells have been initiated [22,23]. For most of these experiments and trials, MSC-BM are prepared with a standard protocol in which nucleated cells are isolated from a bone marrow aspirate with a density gradient and then are enriched and expanded in the presence of FBS by their tight adherence to plastic tissue culture dishes. The MC-L in the present study were cultured in a similar manner based on their adherent property to plastic dishes. Morphologically, these cells looked similar to MSC-BM under the phase contrast microscope. The observation of a decrease in proliferative capacity of cells with increasing passages (rigorous growth at passages 2 and 3, proliferation rate decrease at passage 4 and 5 with slight changes in morphology, and no further proliferation at passage 6) and minor morphological changes in culture have made us restrict our study till passage 6. MC-L cells showed colony forming efficiency of 30%–40% at passage 2, which decreased to 10%–15%, and further with increasing passages. MSC-BM showed similar behavior. The MC-L showed 22.9 population doublings, which was in close approximation to MSC-BM, which showed 30.9 population doublings. This resembles the reported in vitro life span of human MSC-BM (22–23 doublings beginning at primary culture [24] and 15 at passage 1 [25]). Moreover the cultures undergo subtle changes as they expand with a marked decrease in the rate of proliferation and plasticity [25,26] as observed both in MC-L and MSC-BM.

The immunophenotyping of MC-L cells showed a remarkable similarity with the surface antigen profile of MSC-BM as evident from the results of both immunofluorescence and flow cytometry. Like the MSC-BM, the MC-L showed similar expression patterns for CD106 (VCAM), CD54 (ICAM), CD166 (ALCAM), CD90 (Thy-1), CD29 (inetgrin β1), and CD71 (transferrin receptor) markers and negative expression for hematopoietic markers. The data shows a difference in expression patterns of CD105 between MC-L (21.42% ± 4.133) and MSC-BM (71.33% ± 6.66). While the expression levels in MSC-BM are similar to those previously reported by Oswald et al. [27], the present data on the lower expression levels in mesenchymal cells of limbal origin is not sufficient to derive any further conclusions. However, since the endothelial markers (Flt1, Flk1, VE-Cadherin, and CD31 as shown in Table 4) were negative in the limbal-derived mesenchymal cells, we speculate that they are probably neither endothelial derived nor does it point toward endothelial differentiation. We also observed a negative expression profile of MC-L for embryonic stem cell (ESC) markers such as SSEA1, Tra-61, and Tra81, which is in contrast to the observations made by Dravida et al. [8]. This difference could be attributed to the difference in the source of cells such as sorted and unsorted cells.

Similarly, the observation for MC-L made with RT–PCR in which the mesenchymal cells derived from the extended limbal epithelial cultures showed no expression of epithelial markers (such as cytokeratins, p63α, etc.,) or their receptors (EGFR), except for the common intercellular gap junction protein, connexin 43, and glycolytic enzyme, α-enolase. This is similar to what was observed for MSC-BM. We also observed some dissimilarities in expression patterns between MC-L, MSC-BM, and LECs. Similar to MSC-BM, MC-L have shown no expression of PAX−6, which is deviating from the observations of Funderburgh et al. [17] who have shown that an ABCG2-expressing cell population in the corneal stroma also expressed PAX−6, a homeobox gene product not expressed by adult keratocytes. This was probably due to the fact that stromal cells at passage 2 used for RNA isolation could have been showing an adult phenotype. Another difference in expression between the two cell types is the expression of NGF receptor, p75, by MSC-BM and LECs and not by MC-L. The expression and role of p75 in LECs has been well documented [28], and its expression and role in the neuronal differentiation of bone marrow-derived MSC-BM has been recently studied [29]. This difference in expression could be due to the source of these limbal spindle cells from extended limbal epithelial cultures but nevertheless reiterates the MC-L to be of mesenchymal origin.

In this study, we also demonstrated the multilineage differentiation of MC-L into adipocytes and osteocytes, similar to the plasticity of MSC-BM. Also the low level and absence of Major Histocompatability Complex (MHC) class II molecules in MC-L is similar to their levels in MSC-BM (data not reported here). Though the evidence points toward their limbal stromal location, the in vivo role of these cells is not known and extrapolation is beyond the scope of this study. However, a literature review points toward the presence of mesenchymal stromal cells in corneal stroma, similar to our hypothesis. In the study by Choong et al. [30] on keratinocytes isolated from adult human corneal tissues, corneal stromal cells (CSCs), were shown to be CSC^CD13, CD29, CD44, CD56, CD73, CD90, CD105, CD133+/ HLA-DR, CD34, CD117, CD45-^ similar to that of MSC-BM. These cells were also able to differentiate into adipocytes and osteocytes. Yamagami et al. [31] also evaluated for the presence of bone marrow-derived cells in normal human corneal stroma and showed that the CD45-positive cells in the anterior stroma of the central and paracentral cornea and stromal layers of the peripheral cornea also uniformly expressed CD11b, CD11c, CD14, and HLA-DR antigen but not CD3, CD19, CD56, or CD66, which are indicative of bone marrow-derived monocyte lineage cells. They concluded that these cells could play a role in immune responses in the human cornea. An independent study by McCallum et al. [32] phenotypically compared epithelial and non-epithelial components of human corneal and conjunctival microenvironments using a panel of monoclonal antibodies for epithelial cell maturation, mesodermal-derived fibrous tissue and vessels, specific keratins, and MHC class I and II antigens and suggested that the cornea and conjunctiva had similar antigenically defined pathways of maturation.

However, we know that MSC-BM comprise a multifunctional tissue consisting of heterogeneous cell populations that provide a specialized microenvironment for controlling the process of hematopoiesis [33]. The MC-L form part of the niche for limbal stem cells (cells derived from the limbal stroma underlying the limbal epithelium) and did show similarities in the phenotypic profile of MSC-BM i.e., adherent nature, similar surface antigen expression, low immunogenicity and colony forming capability, self-renewal capacity and plasticity (unpublished data). We thus speculate that the niche stromal cells are special cells which might play a role in providing specialized microenvironment in limbal stem cell maintenance. But, its role in diseased and normal states cannot be extrapolated in the current study.

In conclusion, the stromal cell cultures from limbal explants are of stromal origin and fibroblastic in nature and share properties with MSC-BM. Thus, our study shows that the limbal stroma supporting the limbal epithelium possesses a unique population of cells similar to MSC-BM in their culture characteristics, phenotypic marker expression profile, colony forming efficiency, population doubling capacity, and low immunogenicity. However, the role of these cells in vivo and their potential application in vitro needs to be further explored.
